# Mortality selection during the 2003 European heat wave in three-spined sticklebacks: effects of parasites and MHC genotype

**DOI:** 10.1186/1471-2148-8-124

**Published:** 2008-04-30

**Authors:** K Mathias Wegner, Martin Kalbe, Manfred Milinski, Thorsten BH Reusch

**Affiliations:** 1 Department of Integrative Biology (IBZ), Experimental Ecology, ETH Zürich, Universitätstrasse 16, CH – 8092 Zürich, Switzerland; 2 Max-Planck-Institute for Evolutionary Biology, Department of Evolutionary Ecology, August-Thienemann-Str. 2, 24306 Plön, Germany; 3 Institute for Evolution and Biodiversity, Evolutionary Ecology, Westfälische Wilhelms-Universität, Hüfferstrasse 1, 48149 Münster, Germany

## Abstract

**Background:**

Ecological interaction strength may increase under environmental stress including temperature. How such stress enhances and interacts with parasite selection is almost unknown. We studied the importance of resistance genes of the major histocompatibility complex (MHC) class II in 14 families of three-spined sticklebacks *Gasterosteus aculeatus *exposed to their natural macroparasites in field enclosures in the extreme summer of 2003.

**Results:**

After a mass die-off during the 2003-European heat wave killing 78% of 277 experimental fish, we found strong differences in survival among and within families. In families with higher average parasite load fewer individuals survived. Multivariate analysis revealed that the composition of the infecting parasite fauna was family specific. Within families, individuals with an intermediate number of MHC class IIB sequence variants survived best and had the lowest parasite load among survivors, suggesting a direct functional link between MHC diversity and fitness. The within family MHC effects were, however, small compared to between family effects, suggesting that other genetic components or non-genetic effects were also important.

**Conclusion:**

The correlation between parasite load and mortality that we found at both individual and family level might have appeared only in the extraordinary heatwave of 2003. Due to global warming the frequency of extreme climatic events is predicted to increase, which might intensify costs of parasitism and enhance selection on immune genes.

## Background

Most organisms are infected by a multitude of parasites species [1]. The associated fitness costs for hosts are often profound [2], rendering the host-parasite relationship as one of the most intense ecological interactions. The selection pressure on the host to overcome infection might result in an evolutionary arms race between parasite and host. One consequence of such host-parasite co-evolution is the maintenance of genetic diversity in defense genes resulting in marked fitness differences among host genotypes [3,4]. The identification of variable genetic loci that determine specific resistance and thereby enhance host fitness is of prime importance in ecological and evolutionary research [5]. The most striking example of genetic polymorphism that is maintained by parasites is the vertebrate major histocompatibility complex (MHC). MHC molecules activate T-cells by presenting parasite-derived peptides, if the host's individual collection of MHC molecules includes those that can bind to a peptide of the actual infectious agent. Otherwise the infection escapes a T cell response. This suggests that pathogens are the ultimate cause driving MHC diversification [6] and several studies found associations between resistance and presence of single MHC alleles or MHC haplotypes (reviewed in [7-11]).

This applies to three-spined sticklebacks *Gasterosteus aculeatus *L. MHC class II loci that were shown to influence parasite load [12-14] and female mate choice [15,16]. Interestingly, these studies, along with findings from sparrows [17], turkeys [18] and pythons [19] demonstrate that an intermediate number of MHC sequence variants may be favored by selection, as has been predicted by an optimality model [20]. The crucial evidence that is missing thus far is the link between an intermediate number of MHC alleles and maximal Darwinian fitness, under natural, yet still controlled settings. Hence, we wanted to combine advantages of a field study with the knowledge from earlier experimental studies [13,14] by using enclosures in the field. Enclosures were stocked with 14 lab reared, parasite free fish families.

Based on previous laboratory studies [13,14], we predicted that genotypes with an intermediate number of MHC class IIB sequence variants are least infected under more natural field conditions and thus may have higher survival rates. Such a result would reveal the still missing direct link between the number of MHC sequence variants and fitness under almost natural conditions.

Recent studies suggested that a habitat specific MHC genotype cannot fully account for local adaptation to the sympatrically prevalent parasite fauna. The family specific genetic background still explained a large proportion of parasite load [21]. Since parasite load is dependent on individual MHC diversity in sticklebacks [13,12] a true genetic family background effect can best be assessed in families that lack variation in individual MHC diversity. Therefore, we wanted to test the relative importance of MHC genotypes compared to other genetic components by using two kinds of families: one group of families with variable MHC genotypes of the same kind used in previous studies (i.e. segregating families [22,13,23] and one where families only showed a single MHC genotype (non-segregating families). The latter kind results from a cross of parents, which are homozygous at their MHC loci, while segregation of MHC genotypes results from at least some loci being heterozygous. A comparison of both types of families can separate effects attributable to variation in individual MHC diversity in segregating families from effects only dependent on the family genetic background in non-segregating families. If individual MHC diversity is responsible for determining parasite load within-family variation as well as among family variation should be larger for segregating families.

It has been shown recently that the expression of virulence in coupled genetic interactions between hosts and parasites depend on abiotic factors like temperature [24-27]. The strength of selection on MHC diversification might be amplified by additional environmental stress [28]. In which direction expression of virulence is modified is however hard to predict [26]. Hence, phenomena associated with global climate change such as heat waves or precipitation extremes will alter the impact of parasitism, ultimately selecting for increased genetic variability at immune defense loci such as the MHC. On a larger geographic scale MHC diversity was already shown to covary with temperature and bacterial diversity in the water body among Canadian populations of salmon [29]. Our study conducted in 2003 coincided with a period of exceptionally high temperature in central and northern Europe that may be viewed as precursor of future climate extremes [30]. High water temperatures led to substantial mortality in our experimental population. We explore here whether parasitism was a likely cause of mortality selection, and whether this is linked to MHC diversity on a local scale as well.

## Methods

### Fish breeding

In spring 2003 enclosure cages were stocked with fish from 14 lab-reared families. We chose two types of families on basis of their MHC genotypes (for detailed methods see Wegner et al. [13]). In order to elucidate the role of chromosomal segregation, parents that were homozygous at MHC class II loci sired four families. While the MHC class IIB genotypes differed between the four families, all offspring within one such a family have the same MHC genotype (Table 1). Members of the remaining 10 families differed in number and composition of MHC class IIB sequence variants, possessing between three and nine different MHC class IIB sequence variants (Table 1). Within these families genotypes segregated according to Mendelian expectations (strongest deviation in family 13, χ^2^_(df = 3) _= 6, *P *= 0.112). Stickleback MHC class II genes form a linkage group of presumably up to 6 paralogous copies [31] that originated by recent duplication, making it impossible to analyze separate loci [32,33]. After spawning the eggs were removed from the nest and kept in aeriated glass jars (1.5 l) until hatching. Hatched fry was then transferred to 16 l aquaria and later separated to form groups of 20 – 30 fish per tank. Segregating and non-segregating families were randomly interspersed into aquaria and at the start of the experiment each family contributed 20 individuals, except for family 7 where only 18 individuals were available. Before stocking fish were weighed, measured and part of a spine was clipped for DNA extraction.

**Table 1:** Characteristics of the 14 families of three-spined sticklebacks.

FAMILY (hatching date)	MHC GENOTYPE^1^		N^2^		PARASITE LOAD^3^
1 (06.06. 2002)	Segregating (5,8)	M89b, M90, M92, S89b, S91	5/11	8	0.40 ± 1.01
		M89, M92, M92–93, S89, S92			
		M89, M89b, M92, M92–93, S89, S89b, S91, S92	3/7		
2 (07.06. 2002)	Segregating (4,5,6,8)	M89b, M92–93, S89, S92	2/7	6	0.09 ± 0.94
		M89c, M90, M92, S89b, S91	2/5		
		M89b, M89c, M90, M92–93, S89, S92	1/3		
		M89b, M90, M92, M92–93, S89, S89b, S91, S92	1/4		
3 (28.04. 2002)	Non-segregating (4)	M90, M92, S89b, S91		11	-0.50 ± 1.61
4 (05.05. 2002)	Non-segregating (4)	M90, M92, S89b, S91		7	0.46 ± 2.04
5 (21.05. 2002)	Non-segregating (4)	M89, M93, S89, S91		9	-0.69 ± 1.67
6 (15.05. 2002)	Segregating (4,8)	M89b, M92–93, S89, S92		0	n.a.
		M89b, M89c, M90, M92–93, S89, S89b, S90, S92			
7 (03.05. 2002)	Segregating (6,7,8)	M90, M92, M92–93, S89, S89b, S91	0/2	1	n.a.
		M89b, M89c, M92, M93, S89, S91, S92	1/9		
		M89b, M89c, M90, M92–93, S89, S89b, S90, S91	0/7		
8 (02.05. 2002)	Non-segregating (4)	M90, M92–93, S89b, S92		5	0.98 ± 1.37
9 (25.04. 2002)	Segregating (3,4,5,6)	M89c, M90, S90	0/4	3	1.98 ± 1.66
		M89, M93, S89, S92	1/6		
		M89c, M90, M91, S90, S91b	1/4		
		M89, M90, M91, S89, S90, S91b	1/5		
10 (02.05. 2002)	Segregating (4,6,7,8)	M90, M92, S90, S91	0/4	1	n.a.
		M89b, M92–93, M93, S89, S90, S91	1/9		
		M89b, M90, M92, M93, S89, S89b, S92	0/2		
		M89b, M90, M92, M92–93, S89, S90, S91, S92	0/5		
11 (04.05. 2002)	Segregating (4,5,7,8)	M89b, M93, S89, S92	0/3	2	1.89 ± 1.36
		M89b, M89c, M91b, S89, S90	1/7		
		M89b, M90, M92, M93, S89, S90, S91b	0/3		
		M89c, M90, M91b, M92, S89, S90, S91b, S92	1/7		
12 (24.04. 2002)	Segregating (5,6,8,9)	M89c, M91b, S89, S90, S93	1/7	1	n.a.
		M89b, M91b, M92–93, S89, S92, S93	0/2		
		M89c, M90, M91b, M91, S89, S90, S91, S92	0/3		
		M89b, M90, M91, M92–93, S89, S90, S91, S92,	0/7		
		S93			
13 (23.04. 2002)	Segregating (3,4,5,6)	M89, M89b, S89	0/2	3	0.56 ± 0.94
		M89, M89b, S89, S93	0/3		
		M89b, M91b, M92–93, S89, S92	2/9		
		M89, M89b, M92–93, S89, S92, S93	1/6		
14	Segregating (4,6,7,8)	M89b, M93, S89, S91	0/3	1	n.a.
		M89b, M92, M93, S89, S91, S92	1/7		
		M89, M89b, M92, M93, S89, S91, S92	0/7		
		M89, M89b, M92, M93, S89, S89b, S91, S92	0/3		

^1 ^Given are the number of sequence variants for each genotype in the left column and the allelic composition as migration speeds in bp of both primer pairs (M + S) used for genotyping in the right column.

^2 ^N: The right column shows the number of identified and dissected survivors out of the 20 fish used per family. The left column shows the survivors as a fraction of the total number of each genotype used at the start of the experiment in segregating families. Order of genotypes corresponds to the MHC genotype column. Different genotypes with identical number of sequence variants have been pooled.

^3 ^Parasite load was measured as the sum of standardized log-transformed infection intensities for all parasite species and family means (± SE) are shown for families with at least two survivors.

N.a. = not applicable

### Enclosures & Dissection

Fish were exposed to their natural macroparasite fauna in enclosure cages in the lake Grosser Plöner See, Germany (10° 25' 50" E, 54° 09' 21" N). We used stainless steel net cages measuring 1 m * 0.5 m * 0.5 m with mesh size of 5 mm as enclosures. The 14 fish families were evenly distributed over all cages (i.e. 4 fish/family and cage) resulting in 55–56 fish per cage. This way, we controlled for cage effects and can assume that all fish within one cage were exposed to a similar number of parasites. On April 30^th ^2003 the cages were placed into lake at a depth of ≈1 m, when fish were between 327 and 372 days old (Tab. 1). Enclosures were checked and cleaned from algae fortnightly to enable immigration of invertebrate intermediate hosts and free-living infective parasite stages into the cage. Extreme water temperatures with a maximum of 24.3°C (compared to a maximum of 19.6°C in the previous year) and associated evaporation during August caused lake water levels to drop dramatically. To prevent oxygen depletion, we moved the cages further into the lake keeping the cages at the same depth. This could, however, not prevent a mass die off, in which 78% of the fish died in early August. The remaining 61 alive fish were brought to the lab on 12^th ^of August to be dissected during the following two weeks. Of the dead fish we found only a few more or less intact corpses. Since dead fish obviously decomposed rather quickly, an accurate determination of parasite load was impossible. Therefore, we dissected only the surviving fish. For counting parasite species we killed the fish in an excess of 3-aminobenzoic acid ethyl ester (MS 222) and followed the dissection protocol used by Kalbe et al. [34].

### Genotyping

We genotyped each fish from the 2003 study at its MHC class IIB loci. To this end we applied single stranded conformation polymorphism (SSCP) on a 124 bp PCR product covering the functionally important antigen binding region of the exon 2 of the MHC class II β chain [35,16]. Individual numbers of MHC class IIB sequence variants ranged from three to nine. Genomic architecture of the stickleback MHC class IIB region is not known in great detail. Genomic screens indicated that up to six MHC class IIB loci might be present [31]. These loci are partly organized in recently duplicated tandem repeats [32] but the distribution of MHC genotypes in the field suggests that the number of loci might actually vary between individuals, which is a situation commonly found in other fish species [36,37]. Over 90% of sequence variants detected this way are transcribed to messenger RNA suggesting functionality [13]. To genetically tag and identify individuals, we also genotyped all fish at the start and all live fish at the end of the experiment using five polymorphic microsatellite loci [38,39] and could identify 58 of the 61 survivors unambiguously. The remaining three fish were left out of the analysis.

### Statistical analysis

All tests were performed in JMP professional 6.0 (SAS Institute), only generalized linear models with negative binomial error distributions were calculated using R statistical package [40]. We performed one analysis on factors determining mortality that included all fish. A second analysis comprised only those individual fish that survived the summer of 2003. Only the latter data set was used for analyzing parasite infection data.

#### Parasite loads

As we were interested in family effects resulting in differences in infecting parasite community, we performed a linear discriminant analysis (lda) using log-transformed parasite counts as response variables and fish family as classification variable. Prior probabilities were set to the observed numbers of dissected fish and the first two axes were kept for visualization.

Given a family specific infection patterns we were further interested in detailed differences in infection intensity for each parasite species. Macroparasitic infections usually follow a negative binomial distribution [41] and we first fitted a negative binomial distribution to determine the aggregation parameter *k *for each parasite species and used this in the subsequent generalized linear model (GLM). Each GLM included family and cage as factors controlling for uneven distributions of parasite stages between cages.

We were also interested in a measure of total infection intensity because infection by multiple parasite species might increase fitness costs. It is, however, likely that the cost inflicted on the host differ between parasite species, with some species causing less harm than others at identical infection intensities. It is fair to assume that the range of infection intensities within each species over all hosts roughly correlates to the costs of infection. Therefore we used the sums of all standardized log-transformed count values from all species as a measure of overall parasite load. This procedure sets the mean for each species to 0 achieving equal contribution of each species to the sum. This measure correlated well with the number of parasite species infecting the host (*R *= 0.656, n = 51, *P *< 0.0001, used by Wegner et al [12]) while also taking the abundance of each single species into account. Total parasite load measured this way followed a normal distribution (Shapiro-Wilk W-test for deviation from normality: *W *= 0.975, *P *= 0.472).

To investigate the role of individual MHC diversity in parasite infection in segregating families we needed to control for cage effects and family specific genetic effects other than MHC genotype. Therefore, we expressed total parasite loads as residuals of a linear model with the standardized sum as response and cage and family as factors. Hypothesizing an optimal intermediate number of MHC class IIB sequences variants, we tested the effect of MHC on these residuals by fitting a quadratic polynomial as the simplest function possessing a minimum.

#### Survival

To examine effects of the number of MHC class IIB sequence variants within segregating families we scaled survival probabilities for each MHC genotype to its respective family mean. This way, any heritable trait influencing mortality that differed among families would not influence the analysis of the main factor of interest, i.e. the number of MHC class IIB sequence variants. If an MHC genotype survived better than the family average it would be assigned a positive score, while an MHC genotype with lower survival would get a negative one. If an intermediate number of MHC class IIB sequence variants is associated with higher survival, we would thus expect a higher proportion of intermediate genotypes with positive values.

## Results

### Among families: Parasite load

**Figure 1:**
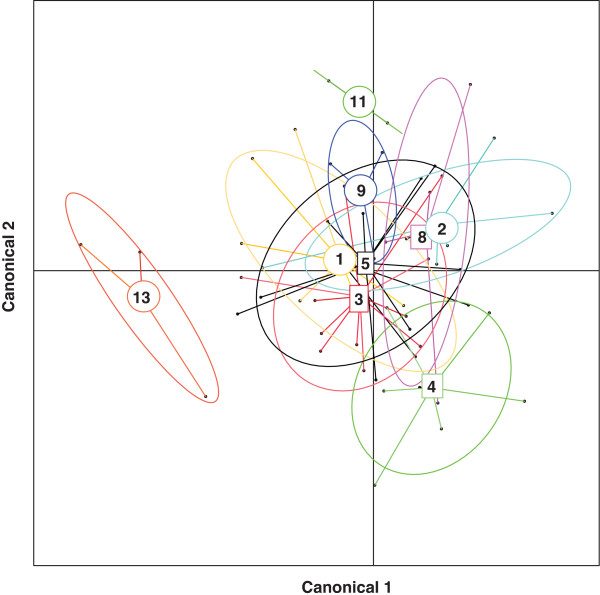
**Ordination of the first two canonical axes derived from the linear discriminant analysis on log-transformed infection intensities of 12 parasite species.** Circles represent group means of segregating families and squares to non-segregating families with single observations connected by lines. 67% of individual fish could be correctly assigned to their original family based on their infecting parasite community (P = 0.037).

**Figure 2:**
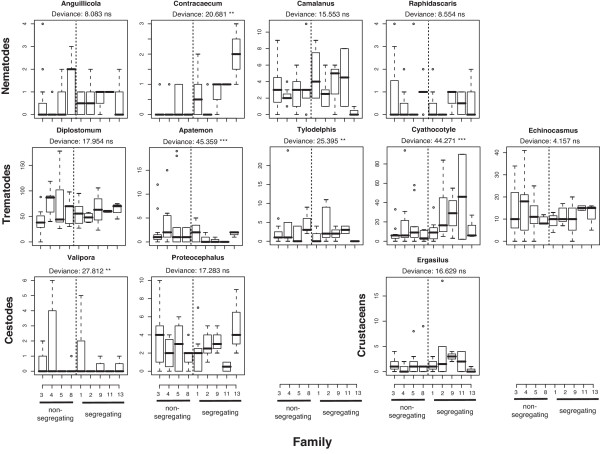
**Infection intensities of twelve macroparasite species in nine three-spined stickleback families with at least two survivors. **Boxplots show medians (black lines), 25–75% quantiles (boxes), 5–95% quantiles (whiskers) and values outside these ranges (circles). The deviance of the family term from a Generalized Linear Model (GLM) with negative binomial error distributions is given for each species. Bonferroni corrected significant differences among families are indicated based on the family deviance: ***: P < 0.001, **: 0.001 < P < 0.01, ns: not significant.

We found a wide range of parasite infection intensities for each of the twelve macro-parasite species surveyed in the surviving fish. The parasite species included were the nematodes *Anguillicola crassus*, *Contracaecum *spec., *Camallanus lacustris *and *Raphidascaris acus*, the digenean trematodes *Diplostomum *spec., *Apatemon cobiditis*, *Tylodelphis clavata*, *Cyathocotyle prussica *and *Echinochasmus *spec., the cestodes *Valipora campylancristrota *and *Proteocephalus filicollis*, as well as the crustacean *Ergasilus *spec These parasite species infect various tissues of sticklebacks ranging from inner organs like eyes and muscles, over the digestive tract (gut and gall bladder) to gills and skin [34]. Except for one fish, which did not get infected at all, all other fish got infected by at least 3 different parasite species. This resulted in simultaneous infection of different organs and most likely affected performance of the fish in several ways [34]. The infection patterns of the parasite species formed communities specific to fish families as indicated by the linear discriminant analysis, which could predict fish family with an overall accuracy of 67% (Fig. 1). This proportion of explained variance was significant in Monte Carlo simulations (P = 0.036, 1000 permutations) as well as in conventional MANOVA analysis using the Pillai-Bartlett statistic (Pillai 2.206, approximate F_96,328 _= 1.301, P = 0.048). The within-family prediction accuracy ranged from 0.333 in family 9 to 1 in family 13. Neither prediction accuracy (χ^2^_d.f. = 1 _= 0.195, P = 0.889) nor ordination distance to the respective family mean families (F_1,52 _= 0.084, P = 0.773) differed significantly between segregating and non-segregating, indicating that variation within segregating families depended only to a minor proportion on different MHC genotypes. Segregating families showed however significantly higher distances from the origin of the ordination (Fig. 1, F_1,52 _= 4.760, P = 0.034) indicating that the parasite fauna found within segregating families deviated more strongly from the population mean than in non-segregating families. The differences among families observed from the multivariate analysis were also reflected in univariate analysis for each parasite species. Significant differences among families in infection intensities could be detected for five out of the twelve species after sequential Bonferroni correction (Fig. 2). These parasite species also gave the highest canonical weights for the lda ordination (Fig. 1), indicating that family specific infection patterns were mainly driven by these five parasite species.

Since most fish were infected by at least three parasite species we were also interested on potential fitness effects of the total parasite load. Total parasite load was negatively correlated to the growth of the fish. Growth was calculated as ln (length at dissection) – ln (length at start). Fish that had a higher total parasite load grew more slowly (*R*^2 ^= 0.355, *F*_1,38 _= 7.836, *P *= 0.008). This effect that was independent of initial length indicating that we actually observed an effect of the parasite and not just preferential infection of smaller fish. When comparing families with segregating and non-segregating MHC genotypes, the mean total parasite load of non-segregating families was not significantly different (F_1,52 _= 0.506, P = 0.480). We also found no significant difference in within-family variation supporting the observations from the multivariate data.

### Among families: Survival

**Figure 3:**
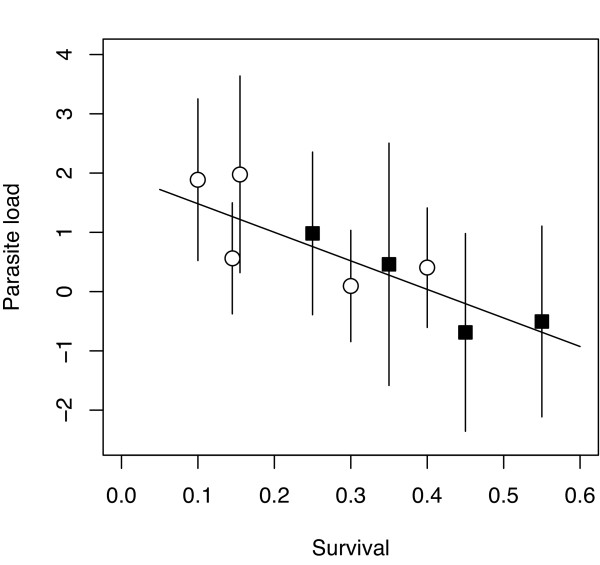
**Correlation between family-wise survival rate and overall parasite load (calculated as the sum of standardized log-transformed infection intensities ± SE).** The regression was calculated on family means weighted by the inverse of the standard errors associated with each family (*R*^2 ^= 0.674, *F*_1,7 _= 14.442, *P *= 0.007). Circles represent segregating families and squares non-segregating families.

There were considerable differences in the proportion of survivors among the families used (Tab. 1, χ^2^_(d.f. = 1, n = 277) _= 42.7, *P *< 0.001). Numbers of survivors ranged from 11 (= 55%) in family 3 to 0 (= 0%) in family 6. Differences in survival were not correlated to differences in family age (*R*^2 ^= 0.031, *F*_1,12 _= 0.348, *P *= 0.567), indicating that age related mortality could be neglected. Among families with at least two survivors there was a negative relationship between proportion of survivors and the overall parasite load averaged over family (Fig. 3, weighted least square regression, *R*^2 ^= 0.674, *F*_1,7 _= 14.442, *P *= 0.007), which could together with the negative correlation to growth rate reflect the increased fitness cost in families with higher average parasite loads. This strong pattern mainly arose out of the combination of all parasite species. While nine out of the twelve parasite species correlated negatively with survival in weighted regressions, only the one for the gill infecting trematode *Echinochasmu*s spec. was actually significant (F_1,8 _= 5.759, P = 0.043), probably representing its role in interfering with oxygen uptake.

### Within families

Since we were mainly interested in partitioning the within family variance according to different MHC genotypes the analyses presented here only include fish from segregating families and focuses on the number of MHC sequence variants within these families.

### Within families: Parasite load

**Figure 4:**
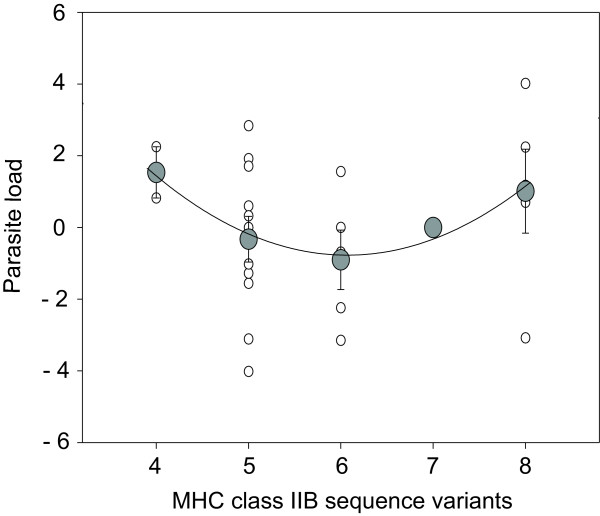
**Mean overall parasite load (± S.E., large circles) and individual parasite loads (small circles) of surviving fish in segregating families.** Parasite load is expressed as GLM residuals with overall parasite load of fish from segregating families as response variable and cage*family as factor. The displayed quadratic polynomial is fitted to the means (*R*^2 ^= 0.9598, *F*_2,2 _= 23.894, *P *= 0.0402). The lowest parasite load can be found at 6.14 MHC class IIB sequence variants, which matches the predicted optimal MHC diversity from previous studies [12-14]. N indicates sample sizes for each allele class.

MHC optimality has so far been demonstrated in terms of parasite resistance among different genotypes of segregating families [13]. In the present study, parasite load could only be estimated in surviving fish. Of 61 survivors only 28 came from segregating families, leaving a very small sample size to analyze patterns of infection across different families. Nevertheless, fish from segregating families with an intermediate number of MHC class IIB sequence variants (i.e. 5–6) tended to have the lowest total parasite load when considering mean values (polynomial regression, *R*^2 ^= 0.960, *F*_2,2 _= 23.894, P = 0.040, minimal parasitation at 6.14 MHC class IIB sequence variants, Fig. 4).

### Within families: Survival

**Figure 5:**
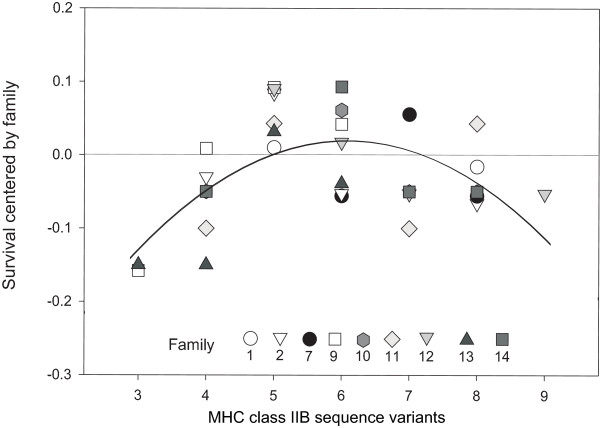
**Relationship between number of MHC class IIB sequence variants and residual survival probability of each MHC genotype.** Note that all fish entered this analysis, i.e. both dead ones reconstructed for their MHC genotype and those that survived. Residual survival probability was calculated as the difference between survival probability if a certain genotype and the overall survival probability of the family. The quadratic polynomial (survival = -0.560 + 0.190 * N_sequence variants _- 0.016 * N_sequence variants_^2^, *R*^2 ^= 0.364, *F*_2,30 _= 8.601, *P *= 0.001) shows that within segregating families fish with a number of 6.086 MHC class IIB sequence variants have highest chance of survival.

The survival probability of each MHC genotype centered by the family mean followed a quadratic polynomial relationship (Fig. 5, survival = -0.560 + 0.190 * N_sequence variants _- 0.016 * N_sequence variants_^2^, *R*^2 ^= 0.364, *F*_2,30 _= 8.601, *P *= 0.001). Separating the parameters of the polynomial reveals that only the quadratic term was significantly different from 0 (t = -3.98, *P *< 0.001) while the intercept and the linear term were not (t_intercept _= -0.45, *P *= 0.658; t_linear _= 1.00, *P *= 0.327). Maximum survival described by this function can be found at 6.086 sequence variants. Since the analysis included all segregating families except family 6 (no survivors), this result may have been biased by inclusion of families that only consisted of a single surviving fish. When these families were excluded from the analysis the result changed only little (*R*^2 ^= 0.487, *F*_2,15 _= 7.116, *P *= 0.007). The same applied to the predicted number of MHC class II variants associated with maximum survival (i.e. 6.18 sequence variants).

## Discussion

This study aimed at disentangling the interaction between parasite-induced mortality selection and individual MHC diversity in a semi-natural setting using outdoor enclosures. Exposing parasite free fish in the field enabled us to evaluate the selective impact of the whole parasite fauna and its relation to individual MHC genotypes (Fig. 4). Furthermore, we were able to identify that mortality selection within families was directly related to the MHC genotype, extending previous studies that found effects on mate choice [15,16] and parasite loads [12-14]. MHC-dependent survival was highest when individuals carried an optimal, i.e. intermediate, number of MHC class IIB sequence variants (Fig. 5).

MHC genotype has been linked to survival in several studies, which measured survival directly [42] or identified protective alleles against fatal forms of disease using fish model systems [43-45]. Similar results have been found in ruminants, where MHC alleles were positively correlated with survival but negatively correlated to intestinal nematode burden [46]. In the light of these studies, a selective role of parasites in determining survival as a result of the MHC genotype of fish hosts seems plausible.

We can only assume infection intensities among those fish that died before sampling the experiment. An extrapolation of parasite load from the survivors to the dead fish (Fig. 4) is justifiable because previous laboratory studies under benign conditions showed similar patterns of parasite infection [13,14]. Under these assumed infection intensities, higher mortality found in sub- and super-optimal MHC genotypes may at least partly be attributed to higher parasite burden. We can however indirectly assess fitness costs in the surviving fish where growth rate negatively correlated with total parasite load. This pattern was independent of length before stocking and therefore only reflects impeded growth in the enclosures. Slowed growth rate of fish with high parasite burden did therefore not result from low growth rates before parasite exposure and the observed change in growth rates may be attributed directly to higher parasite burdens.

By using within family comparisons, we minimized the effect of genetic factors other than those associated with the MHC class IIB region, because the genetic background characteristic for each family cancels out in this analysis. In between-family comparisons, these family background effects turned out to be quite strong and explained a major proportion of variation in survival as well as parasite infection patterns (Fig. 1, Fig. 2). Family background is also the most likely explanation why non-segregating families showed lower mortality than segregating families. We can however not be sure that these family effects are really truly genetic. They could also represent maternal, epigenetic or environmental effects, which have previously been linked to parasite infection in fish [47]. The unknown nature of these family effects strengthens the observed within-family MHC effect, because this is linked to the MHC genotype. The within family MHC effect was however outweighed by the between family effects as no differences in overall parasite load between segregating and non-segregating families could be detected. The different MHC genotypes within segregating families did also not lead to an increased variance in infection intensities in these families, which might be attributed low sample sizes per genotype due to the high mortality in these families. Parasite infection patterns of segregating families deviated stronger from the population mean (Fig. 1, Fig. 2), which might indicate that the higher segregational variance allows escape from the most prevalent parasites representing the population mean.

## Conclusion

Our data from 2003, a year with extreme summer temperatures, suggests that mortality selection can be strong. The correlation between parasite load and survival in both within and among family comparisons indicates that parasites might indeed have played a role for the high mortality rates. Compared to wild caught fish from the same area infection [34] intensities here were much higher in the majority of parasite species observed in this study. Also pilot studies using the same experimental set-up did show reduced parasite burdens as well as lower mortality rates (M. Kalbe, unpublished data). High infection intensities in 2003 may have been a consequence of bad fish condition, increased exposure to parasites [27], or altered expression of virulence resulting from genotype × genotype × environment interactions (G × G × E) [24-26]. Either way, the high parasite infection intensities might be a result of environmental stress, and will further increase selection on MHC and other immune genes. A similar pattern on a larger geographical scale was previously observed for Atlantic salmon in Canada, where allelic richness and selection on antigen binding regions of MHC class II genes increased with increasing temperature and pathogen richness [29]. Here, we can now not only show that within families a link between individual MHC diversity and mortality exists but that also other family specific factors apart from the MHC will play an important role under extreme environmental conditions. Extreme climatic events as the singular event of the 2003 heat wave are predicted to increase [30]. In light of the family specific parasite loads and mortality under such conditions, the interaction of abiotic environmental stress and parasitism [26] will become an increasingly interesting field for addressing questions of natural selection in the context of global warming.

## Authors' contributions

KMW wrote the manuscript and was involved in fish breeding, genotyping, designing and running the experiment, dissecting the fish and analyzing the data. MK was involved in fish breeding, designing and running the experiment, dissecting the fish and writing the manuscript. MM was running the fish breeding and was involved in designing the experiment and writing the manuscript. THBR was involved in designing the experiment, genotyping as well as analyzing the data and writing the manuscript.
